# A Comparative Evaluation of Antimicrobial and Cytotoxic Efficacy of Biosynthesized Silver Nanoparticles and Chemically Synthesized Silver Nanoparticles Against Enterococcus faecalis: An In Vitro Study

**DOI:** 10.7759/cureus.58428

**Published:** 2024-04-16

**Authors:** Neena Chandran, Sindhu Ramesh, Rajeshkumar Shanmugam, Jayalakshmi S

**Affiliations:** 1 Conservative Dentistry and Endodontics, Saveetha Medical College and Hospital, Saveetha Institute of Medical and Technical Sciences, Saveetha University, Chennai, IND; 2 Conservative Dentistry and Endodontics, Saveetha Dental College and Hospitals, Saveetha Institute of Medical and Technical Sciences, Saveetha University, Chennai, IND; 3 Nanobiomedicine, Saveetha Medical College and Hospital, Saveetha Institute of Medical and Technical Sciences, Saveetha University, Chennai, IND

**Keywords:** enterococcus faecalis, azadirachta indica, trisodium citrate, silver nanoparticles, antibacterial activity

## Abstract

Introduction

Effective root canal cleaning and sealing are essential for a successful endodontic procedure. For the purpose of disinfecting root canals, both herbal and non-herbal medications are recommended. This study aimed to analyze the antimicrobial and cytotoxic properties of biosynthesized silver nanoparticles (AgNPs) synthesized from *Azadirachta indica*/neem and chemically synthesized AgNPs from trisodium citrate (TSC) against oral pathogens to be further used as an irrigant in endodontic treatment.

Materials and methods

To synthesize *A. indica *AgNPs, powdered fresh *A. indica* leaves were weighed, added to double distilled water, heated for 30 minutes, and then combined with silver nitrate solution. TSC was also used to create TSC AgNPs. X-ray diffraction (XRD), scanning electron microscopy (SEM), ocular observation, and the ultraviolet-visible light (UV-vis) spectrum were used to characterize the AgNPs. Studies were conducted on the extract's characteristics, including its cytotoxicity and antibacterial activity.

Results

The hue shift and peak on the UV-vis spectrophotometer were signs that AgNPs were forming. The XRD pattern showed that the sample included crystalline AgNPs, mostly spherical ones. By using SEM, the presence of AgNPs was also verified. AgNPs that were synthesized showed antimicrobial efficacy against *Enterococcus* *faecalis*. Compared to chemically synthesized AgNPs, *A. indica* AgNPs showed lower minimum inhibitory concentration (MIC) and minimum bactericidal concentration (MBC) values, a bigger zone of inhibition (ZOI), and less cytotoxic action.

Conclusion

This study demonstrates the minimal cytotoxicity and antibacterial activity of *A. indica* AgNPs against *E. faecalis*. This suggests that they might also be employed as root canal cleaners. Before experimenting with animals or cell lines in clinical trials for endodontic treatment, further research should be done.

## Introduction

Endodontic infections, mainly by *Enterococcus faecalis*, persist despite advancements in treatment techniques due to the bacterium's ability to form biofilms, develop antibiotic resistance, invade dentinal tubules, and thrive within the complex intracanal environment. Complex root structures hinder complete cleaning. Sodium hypochlorite (NaOCl) effectively fights *E. faecalis* but poses tissue safety issues [1]. Exploring alternative irrigation methods is crucial to balance strong germ-killing abilities with tissue safety in endodontic therapy [2]. Achieving comprehensive disinfection remains a challenge, urging modified protocols or new solutions for optimal antimicrobial impact without harming tissues [3]. Balancing potent antimicrobial action and tissue compatibility is the key to achieving better outcomes against persistent *E. faecalis* infections in root canals [4].

Silver nanoparticles (AgNPs) especially those synthesized using trisodium citrate (TSC) or neem/*Azadirachta indica* extracts showcase remarkable antibacterial properties [5]. Chemical synthesis with TSC influences AgNPs' size and attributes, while *A. indica* compounds contribute to its antibacterial nature in green synthesis methods [6]. These eco-friendly approaches create biocompatible AgNPs. Leveraging green synthesis aligns with sustainability and broadens nanotechnology's applications [6]. Despite this, there is a gap in understanding their efficacy in endodontics against *E. faecalis* [7]. This study aims to compare *A. indica* AgNPs to TSC AgNPs, evaluating their antimicrobial and cytotoxic effectiveness specifically against *E. faecalis*, addressing this crucial research gap [8].

*E. faecalis*, renowned for its tenacity and endurance within endodontic environments, poses a significant challenge during root canal treatments. Moreover, the coexistence of other microorganisms such as *Candida albicans *and *Klebsiella pneumoniae* further complicates these infections, highlighting the pressing need for novel and potent intracanal irrigants [8]. The assessment of their antimicrobial efficacy, along with cytotoxicity evaluations, aims to ascertain their safety and effectiveness as a potential therapeutic option in root canal treatments. Bridging this gap in research could pave the way for innovative strategies harnessing plant-based nanoparticle synthesis for enhanced endodontic therapies targeting resilient microbial infections. In order to precisely target *E. faecalis*, the objective of this work is to compare the antibacterial and cytotoxic efficacy of biosynthesized AgNPs (*A. indica* AgNPs) with chemically synthesized TSC AgNPs.

## Materials and methods

The two main phases of this in vitro study were the production of AgNPs and the assessment of their antibacterial activity and cytotoxicity against *E. faecalis*. For the synthesis process, fresh *A. indica* leaves were procured from Chennai, India. Analytical-grade chemicals were purchased from Sigma Aldrich Co. (St Louis, MO, USA).

Synthesis of plant extract

The plant extract was synthesized by a multi-step process. *A. indica* leaves were thoroughly cleaned with tap water and then rinsed with distilled water to remove any foreign matter that could prevent silver ions from adhering during synthesis. Following three days of air drying at room temperature, the leaves were precisely weighed and then coarsely crushed. After being heated to 55°C for 15 minutes, 1 g of this powder was mixed with 100 mL of distilled water, allowed to cool, and then filtered through Whatman No. 1 filter paper. The resultant filtrate was kept for further production of AgNPs at 4°C [9].

Synthesis of silver nanoparticles

AgNPs were synthesized from 0.1 M silver nitrate solutions. *A. indica* AgNPs synthesis involved mixing 80 mL of silver nitrate with 20 mL of plant extract suspension, while TSC AgNPs synthesis combined 1 mg silver nitrate with 70 mL water and 3 g TSC in 30 mL water. Both processes ran on a magnetic stirrer for two days, maintaining homogeneity. Ultraviolet-visible light (UV-vis) spectra scanning tracked color changes indicating silver ion reduction. Synthesized AgNPs underwent centrifugation, and dilutions were made for subsequent analysis, storing them at 4°C in sealed containers [9].

Characterization of silver nanoparticles

Cutting-edge analytical techniques were deployed for a comprehensive characterization of AgNPs. UV-vis spectroscopy tracked the production of AgNPs, monitoring silver ion reduction. The resulting solution was analyzed in a cuvette using an ELICO SL 210 UV-vis spectrophotometer (Elico Ltd., Hyderabad, India), revealing an absorbance peak at 300-700 nm, indicating surface plasmon resonance (SPR) of emerging AgNPs. Scanning electron microscope (SEM) analysis, employing a JEOL MODEL FE-SEM IT800 (JEOL Ltd., Akishima, Japan), offered intricate insights into nanoparticle morphology. Samples were mounted on brass stubs, coated in platinum, and imaged at various magnifications, unveiling morphology, surface topography, and size distribution. X-ray diffraction (XRD) research, utilizing a Bruker D8 Advance X-ray diffractometer, examined structural features. Copper kα radiation at 1.5406 Å with 2θ ranging from five to 90 degrees provided data on crystalline structure, crystal size, and orientation, confirming the presence of metallic silver and elucidating atomic arrangement. The combined use of UV-vis spectroscopy, SEM imaging, and XRD analysis comprehensively characterized AgNPs, unveiling optical properties, morphological features, crystalline structure, and size distribution. These insights pave the way for potential applications across scientific and technological domains.

Bacterial strains

Gram-positive *E. faecalis* ATCC 29212 strains, sourced from Saveetha Dental College's Nanobiomedicine Lab, were cultivated on nutrient agar plates at 37°C for 24 hours using the spread plate technique. Subsequently, the strains were grown on Mueller-Hinton agar plates. Three methods, namely, minimum bactericidal concentration (MBC), minimum inhibitory concentration (MIC), and cytotoxicity tests in culture medium and growth inhibition zone diameters, were used to evaluate antibacterial effects across varying solution concentrations.

Antimicrobial susceptibility test

The antibacterial activity of the produced AgNPs was measured using the agar well diffusion method for the antimicrobial susceptibility test. The base medium was Mueller-Hinton agar, which was prepared and sterilized at 121°C for 15 minutes. This mixture was poured onto sterilized plates and let to harden. Next, sterile polystyrene tips were used to make 9 mm diameter wells, and swabs were used to evenly distribute the test organisms around the agar surface. The produced *A. indica* AgNPs and TSC AgNPs were added to three successive wells on separate agar plates at varied concentrations (25 µL, 50 µL, and 100 µL). The fourth well held a positive control as NaOCl, and the fifth well held a negative control as saline. For 24 hours, the plates were incubated at 37°C. The amount of bacterial growth inhibition surrounding each well was evaluated by measuring and observing the ZOIs after incubation. Using this technique, the antibacterial activity of TSC AgNPs and *A. indica* AgNPs against *E. faecalis* may be compared, revealing information about their potential as antimicrobial agents to treat bacterial infections.

Determination of minimal inhibitory concentration

MIC determination assesses an organism's susceptibility to antimicrobial drugs by observing bacterial growth in microtitration plate wells with varying drug dilutions. The MIC represents the drug concentration inhibiting microorganism growth. Prepared Muller-Hinton broth (6 mL) was added to three test tubes, each containing a bacterial suspension (5×10^5^ CF U/mL). Different volumes (25 µL, 50 µL, and 100 µL) of *A. indica* and TSC AgNPs were added to specific wells, while saline and NaOCl served as negative and positive controls in other wells. Incubation at 37°C for up to five hours occurred in batches, followed by evaluating *E. faecalis* growth suppression via the broth's optical density at 600 nm.

Determination of minimum bactericidal concentration

The MBC is the lowest concentration of antimicrobial agent needed to destroy 99.9% of the original inoculum following a 24-hour incubation period under specified circumstances. Following broth microdilution, the MBC was determined by sub-culturing samples from wells or tubes onto nonselective agar plates and incubating them for 24 hours to assess microbial growth. The agar well diffusion method was employed to assess the antibacterial effectiveness of AgNPs on Muller-Hinton agar. After the agar was ready, sanitized, and transferred onto sterile Petri dishes, it was left to solidify. Molten Muller-Hinton agar was filled into Petri dishes, and the bacterium was added. On the solidified agar, uniformly sized wells were created. Following incubation, the plates were examined for the formation of colonies to calculate the MBC value in the absence of any appreciable growth.

Cytotoxicity test

The brine shrimp lethality assay was also used to assess cytotoxicity. After dissolving iodine-free salt in distilled water, saline water was applied to enzyme-linked immunoassay (ELISA) plates. The larval brine shrimp or nauplii were gradually added to every well. After a 24-hour incubation period, the nanoparticles were introduced at different concentrations, and the cytotoxicity was calculated by counting the live nauplii using the following formula: (number of dead nauplii/number of dead nauplii+number of live nauplii)×100. These methodologies allow for a comprehensive assessment of the antimicrobial efficacy of AgNPs against *E. faecalis*, determining their MIC, MBC, and potential cytotoxic effects, essential for evaluating their suitability as antimicrobial agents in biomedical applications.

## Results

Silver nanoparticle characterization

**Figure 1:**
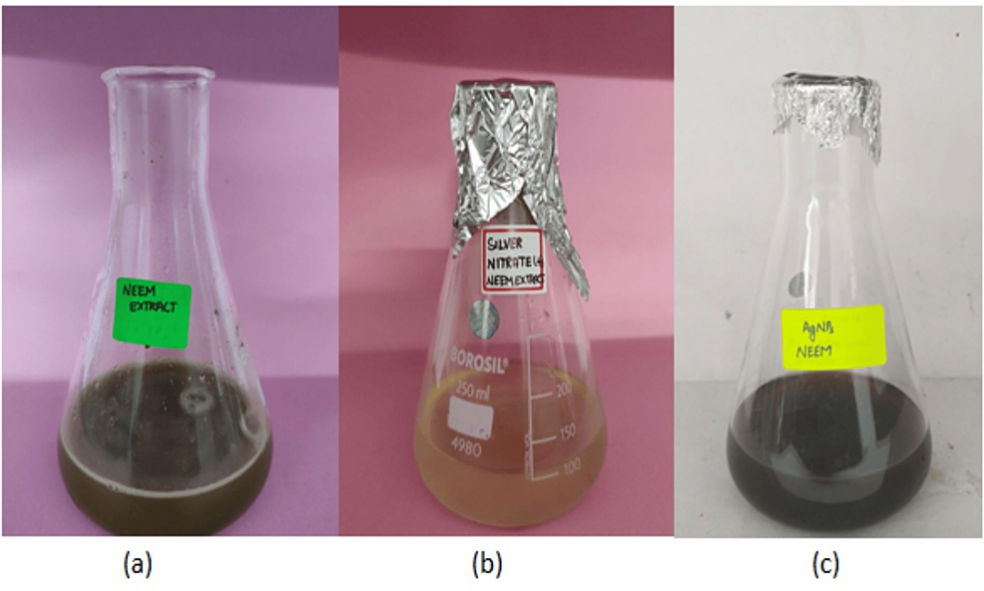
(a) Azadirachta indica extract, (b) mixture of A. indica extract with silver nitrate (before 24 hours), and (c) reduction of silver nitrate (after 24 hours)

**Figure 2:**
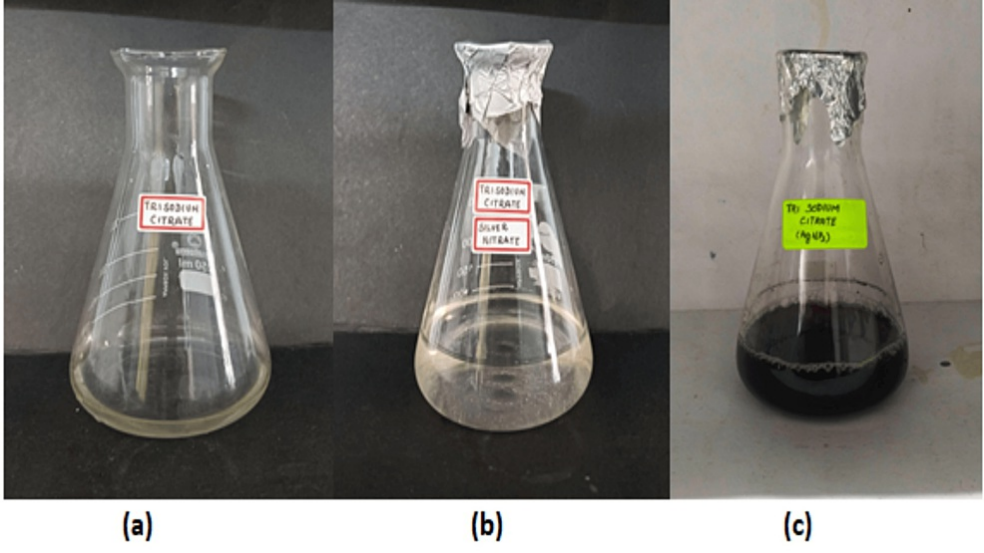
(a) TSC extract, (b) mixture of TSC extract with silver nitrate (before 24 hours), and (c) reduction of silver nitrate (after 24 hours) TSC: trisodium citrate

AgNPs were synthesized by reducing silver nitrate using a blend of manufactured chemicals and *A. indica* leaf extract. Distinct color changes validated successful synthesis of AgNPs as shown in Figures 1-2. These color changes are important markers of the decrease in silver ions and the consequent synthesis of AgNPs. The color change is correlated with the stimulation of SPR, which is a characteristic of nanoparticles. This confirms that the manufacture of AgNPs using *A. indica* leaf extract and chemical procedures is legitimate. These findings underscore the effectiveness of both approaches in producing AgNPs, setting the stage for potential medical applications owing to the unique properties of these synthesized nanoparticles.

Ultraviolet-visible light analysis

UV-vis spectroscopy is crucial for characterizing synthesized nanoparticles, especially AgNPs. Their distinct optical properties interact with specific light wavelengths, showcasing SPR. In AgNPs, aligned conduction and valence bands allow unhindered electron movement, inducing SPR absorption at around 440 nm for *A. indica-*based AgNPs and 435-445 nm for chemically produced AgNPs. This peak, consistent for 2-100 nm metal nanoparticles, aligns with observed color changes during synthesis, validating successful AgNPs' formation from both *A. indica *extract and TSC. AgNPs' absorption, influenced by factors like particle size and chemical environment, demonstrates the robustness of UV-vis spectroscopy in nanoparticle characterization.

**Figure 3:**
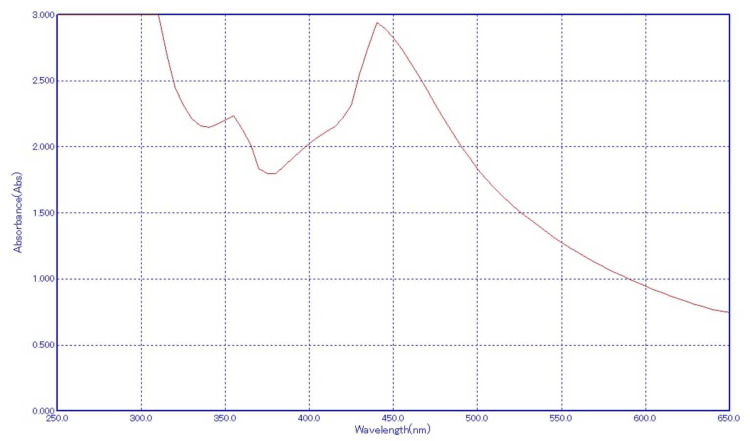
UV-vis of Azadirachta indica AgNPs UV-vis: ultraviolet-visible light;AgNPs: silver nanoparticles

**Figure 4:**
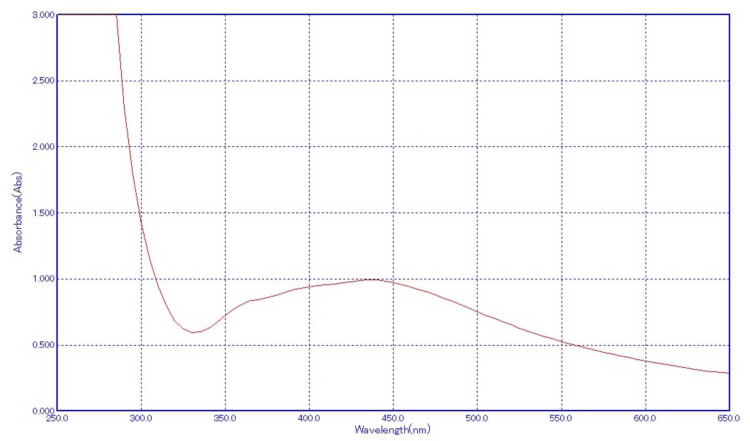
UV-vis of TSC AgNPs UV-vis: ultraviolet-visible light; TSC: trisodium citrate; AgNPs: silver nanoparticles

Figures 3-4 illustrate the UV-vis spectra of chemically synthesized AgNPs and *A. indica* synthesized AgNPs, respectively, showcasing the distinctive absorption peaks that validate the presence of SPR characteristics of AgNPs synthesized via different routes.

Scanning electron microscopy study 

**Figure 5:**
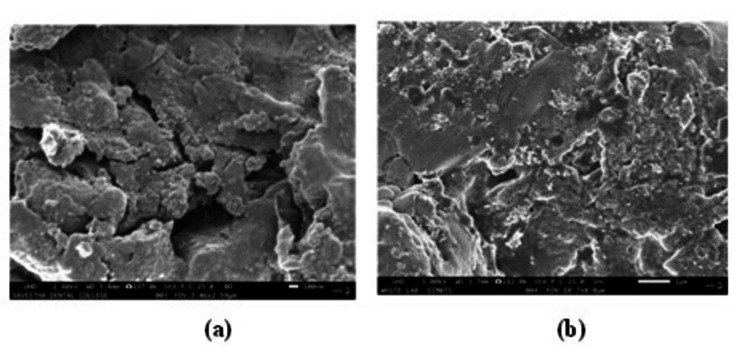
SEM analysis of (a) Azadirachta indica AgNPs and (b) TSC AgNPs SEM: scanning electron microscope; AgNPs: silver nanoparticles; TSC: trisodium citrate

Using JEOL MODEL 6390 equipment and SEM grids, the size and shape of AgNPs were evaluated. The SEM analysis of *A. indica* AgNPs (Figure 5a) and TSC AgNPs (Figure 5b) revealed essential characteristics. The study revealed uniform spherical shapes for AgNPs, measuring 29-60 nm, verified by SEM images. Both *A. indica-* and TSC-synthesized AgNPs exhibited consistent morphology and inter-particle distances, elucidating size distribution within this range.

X-ray diffraction study

**Figure 6:**
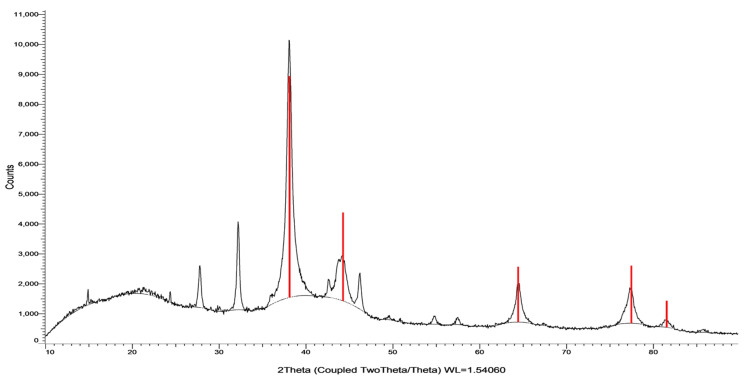
Phase-matched XRD pattern of Azadirachta indica AgNPs XRD: X-ray diffraction; AgNPs: silver nanoparticles

**Figure 7:**
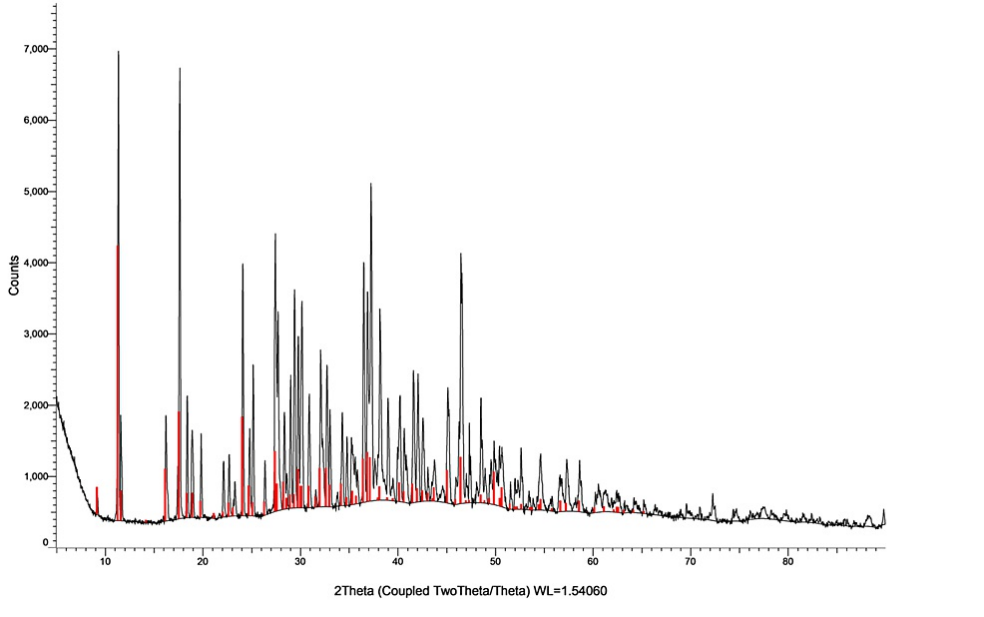
Phase-matched XRD pattern of TSC AgNPs XRD: X-ray diffraction; TSC: trisodium citrate; AgNPs: silver nanoparticles

XRD analysis depicted in Figures 6-7 revealed Bragg reflections at 2θ angles (32.9, 46.9, and 28.5), confirming the silver colloid presence and a face-centered-cubic (FCC) structure. Figures 6-7 showcased phase-matched XRD patterns, reinforcing *A. indica* AgNPs crystallinity and FCC structure. This comprehensive XRD examination definitively validated the crystalline nature of synthesized AgNPs, affirming their successful creation and elucidating structural characteristics.

Antibacterial actions 

**Figure 8:**
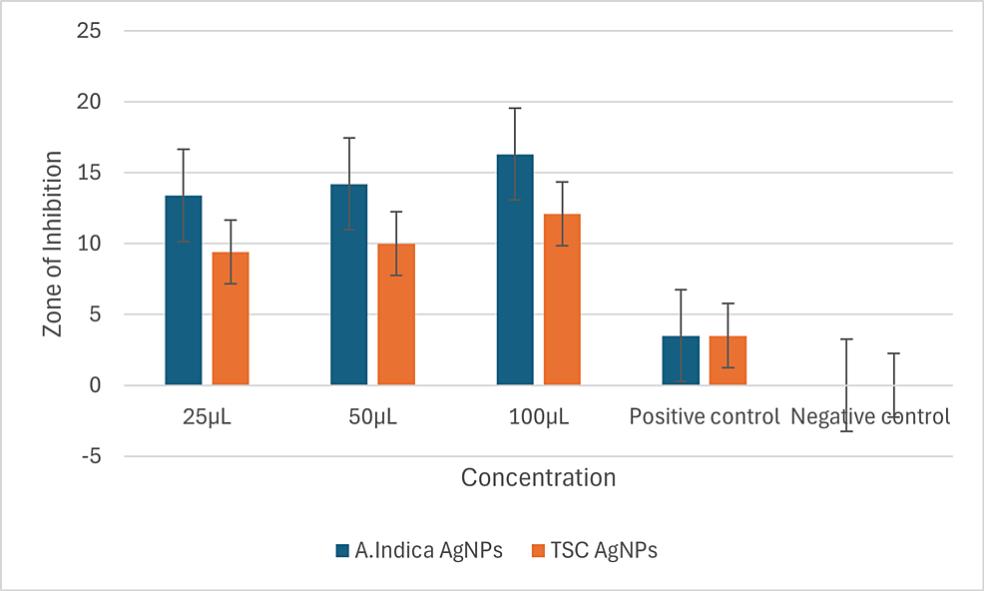
Depicts the antibacterial action of Azadirachta indica AgNPs and TSC AgNPs at different concentrations against Enterococcus faecalis AgNPs: silver nanoparticles; TSC: trisodium citrate

The zone of inhibition (ZOI) test demonstrated concentration-dependent antibacterial effects of synthesized AgNPs against *E. faecalis*. *A. indica* AgNPs exhibited the highest ZOI at 100 μg/mL (16.3 mm), surpassing TSC AgNPs at the same concentration (12.1 mm). At 25 μg/mL, *A. indica* AgNPs also outperformed TSC AgNPs (13.4 mm vs. 9.4 mm). Figure 8 illustrates the ZOI of *A. indica* and TSC AgNPs against *E. faecalis* at varying concentrations, highlighting *A. indica* AgNPs' superior antibacterial action at higher concentrations. This analysis underscores the concentration-dependent antibacterial efficacy of synthesized AgNPs against *E. faecalis*.

**Figure 9:**
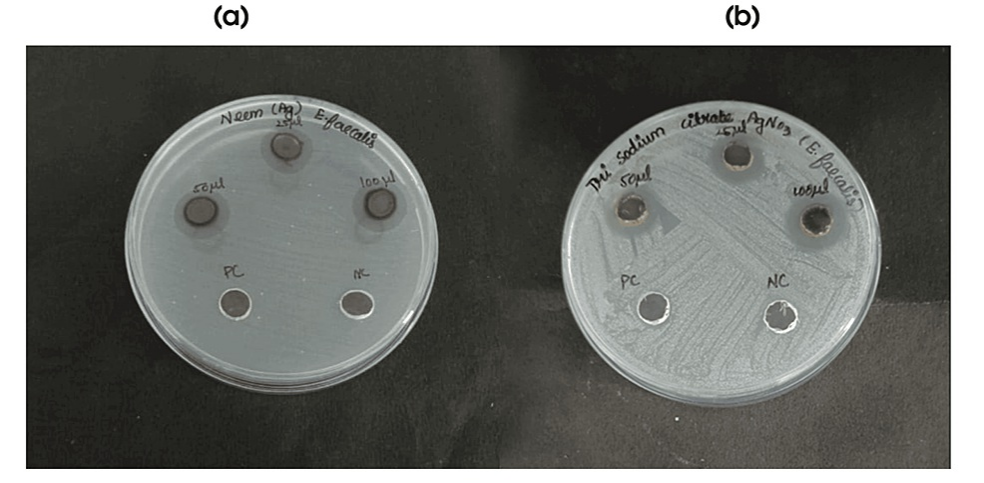
ZOI of (a) Azadirachta indica AgNPs and (b) TSC AgNPs at different concentrations against Enterococcus faecalis AgNPs: silver nanoparticles; TSC: trisodium citrate; PC: positive control; NC: negative control; ZOI: zone of inhibition

Table 1 exhibits ZOI around *E. faecalis* treated with varying concentrations (25 µg/mL, 50 µg/mL, and 100 µg/mL) of *A. indica* and TSC AgNPs, showcasing dose-dependent inhibition.* A. indica* AgNPs displayed larger zones than TSC AgNPs, suggesting stronger antibacterial potency. Positive controls confirmed efficacy compared to the negative control's absence of inhibition. Figure 9 illustrates the agar well diffusion assay test to determine the antibacterial efficacy of both *A. indica* AgNPs and TSC AgNPs at different concentrations.

**Table 1:** ZOI around Enterococcus faecalis on treatment with Azadirachta indica AgNPs and TSC AgNPs under various concentrations AgNPs: silver nanoparticles; TSC: trisodium citrate; ZOI, zone of inhibition

Samples (mm)	25 µL	50 µL	100 µL	Positive control	Negative control
*A. indica* AgNPs	13.4	14.2	16.3	3.5	0
TSC AgNPs	9.4	10	12.1	3.5	0

**Figure 10:**
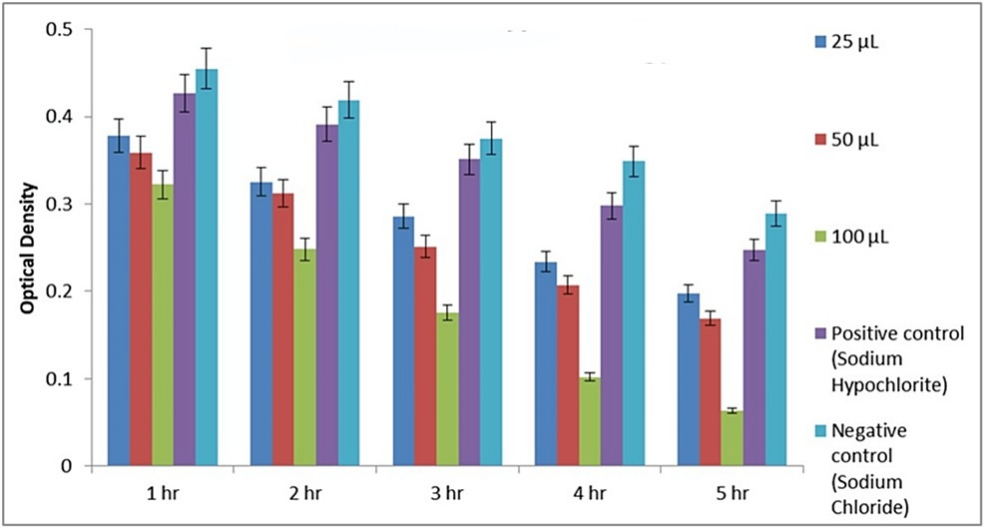
MIC of Azadirachta indica AgNPs MIC: minimum inhibitory concentration; AgNPs: silver nanoparticles

**Figure 11:**
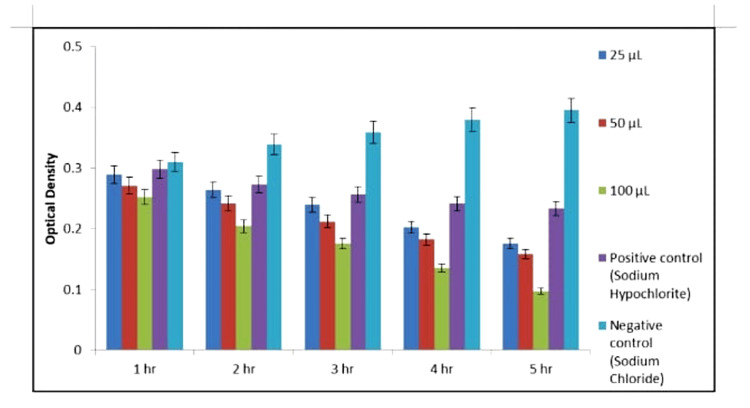
MIC of TSC AgNPs MIC: minimum inhibitory concentration; TSC: trisodium citrate; AgNPs: silver nanoparticles

Figures 10-11 depict MIC values of *A. indica* AgNPs and TSC AgNPs against *E. faecalis* at different concentrations over five hours, determined via the broth dilution method in a 96-well microtiter plate. Consistently low MIC values (100 µg/mL) for both types of AgNPs at all time points demonstrate potent inhibition of *E. faecalis* growth. In contrast, the distilled water control showed higher MIC values, indicating inefficacy. This comprehensive assessment reaffirms the robust antibacterial efficacy of* A. indica* AgNPs and TSC AgNPs against *E. faecalis* across concentrations and time intervals. 

**Figure 12:**
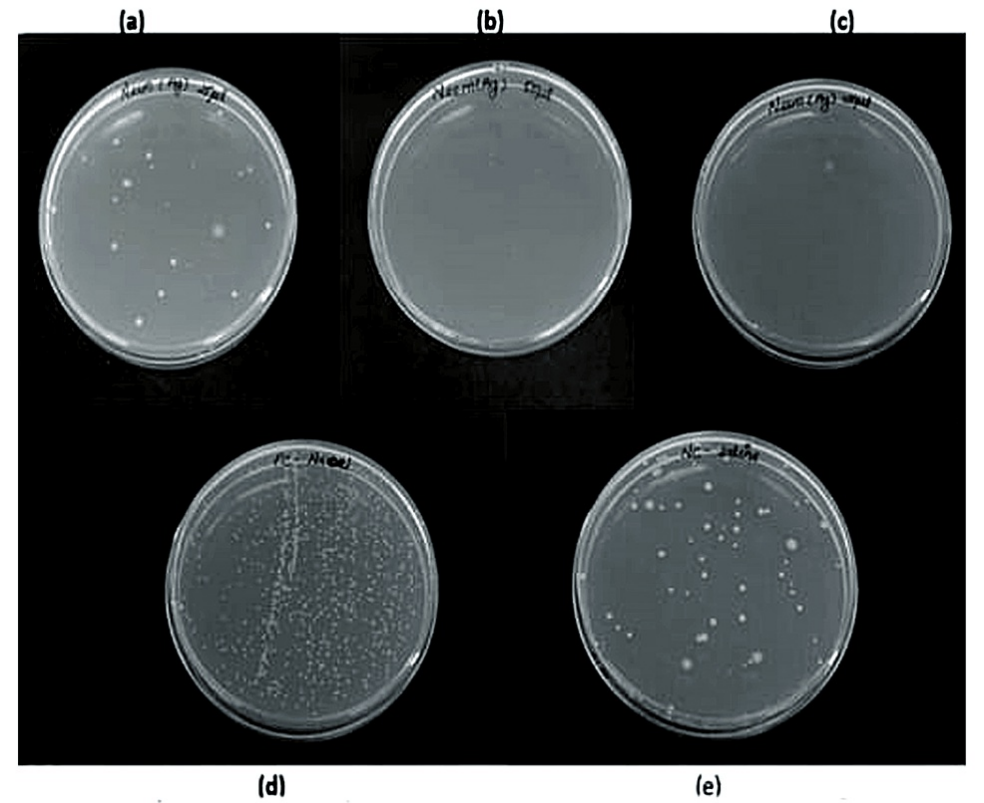
MBC of Azadirachta indica AgNPs at (a) 25 μL, (b) 50 μL, (c) 100 μL, (d) positive control, and (e) negative control MBC: minimum bactericidal concentration; AgNPs: silver nanoparticles

**Figure 13:**
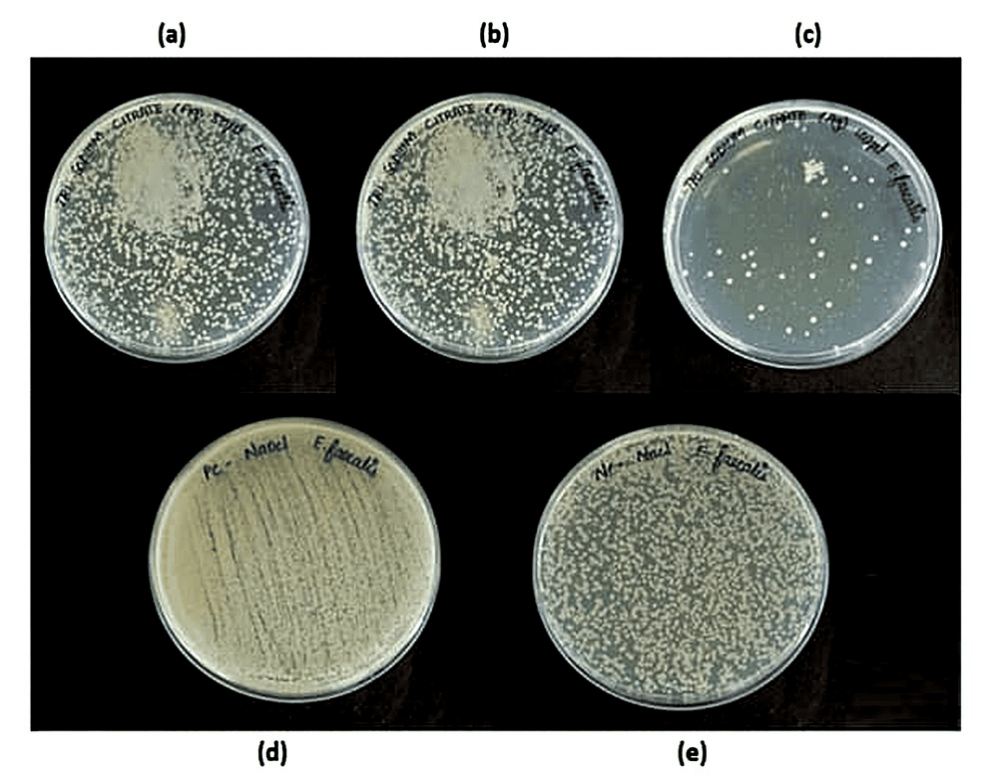
MBC of TSC AgNPs at (a) 25 μL, (b) 50 μL, (c) 100 μL, (d) positive control, and (e) negative control MBC: minimum bactericidal concentration; TSC: trisodium citrate; AgNPs: silver nanoparticles

MBC was computed using the mean colony count (107 CFU/mL) for both groups in order to assess the antibacterial activity against *E. faecalis*. As can be seen in Table 2, adding AgNPs to *A. indica* extract and TSC resulted in varying degrees of bacterial growth suppression as shown in Figures 12-13. The CFU count for *A. indica* AgNPs at 100 µg/mL concentration was significantly lower than that of the other samples, showing that the antimicrobial agent was successful in preventing bacterial growth at this concentration. Since no antimicrobial agent was applied in the negative control sample, the negative control had the highest CFU count.

**Table 2:** MBC of Azadirachta indica AgNPs and TSC AgNPs MBC: minimum bactericidal concentration; AgNPs: silver nanoparticles; TSC: trisodium citrate

AgNPs (mm)	25 μL	50 μL	100 μL	Positive control	Negative control
*A. indica* AgNPs	35×10^2 ^CFU/mL	35×10^2 ^CFU/mL	17×10^3 ^CFU/mL	287×10^4 ^CFU/mL	65×10^4 ^CFU/mL
TSC AgNPs	Enormous growth	Enormous growth	Enormous growth	Enormous growth	Enormous growth

Cytotoxicity

**Figure 14:**
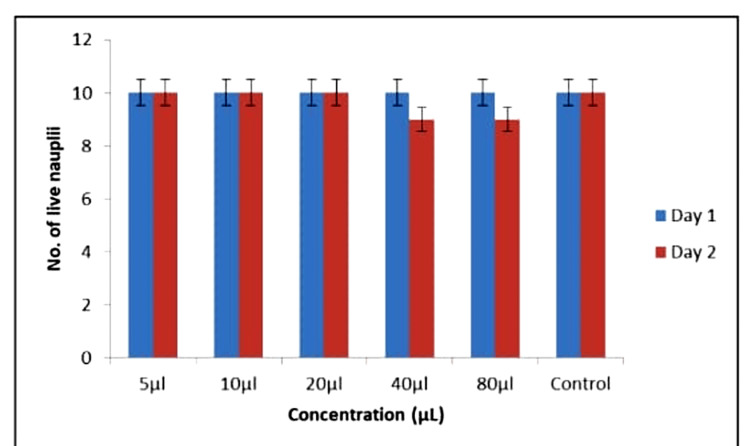
Cytotoxicity activity among Azadirachta indica AgNPs AgNPs: silver nanoparticles

**Figure 15:**
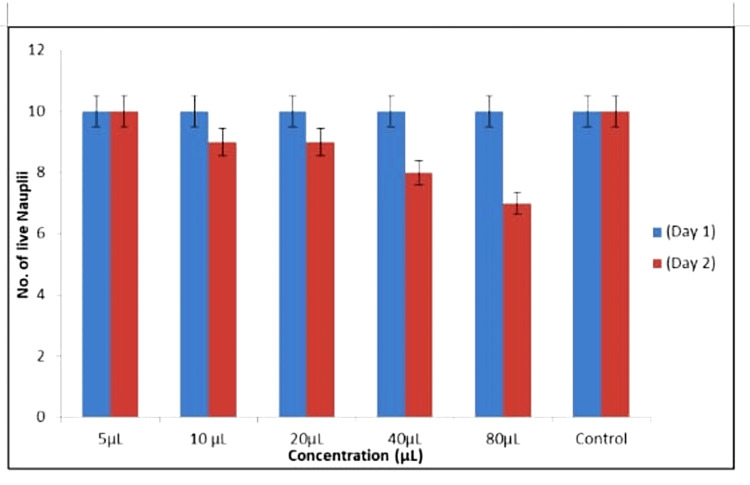
Cytotoxicity activity among TSC AgNPs TSC: trisodium citrate; AgNPs: silver nanoparticles

The experiment monitored nauplii response to *A. indica *AgNPs and TSC AgNPs over 24 hours. At 5-20 μL concentrations, both types of AgNPs sustained nauplii survival initially. Yet, at 40-80 μL, *A. indica* AgNPs had nine surviving nauplii, while TSC AgNPs had seven at 80 μL. Figures 14-15 show cytotoxicity effects. *A. indica* AgNPs demonstrated lower cytotoxicity, preserving nauplii viability at higher concentrations, contrasting with TSC AgNPs' higher toxicity.

## Discussion

In modern dentistry, natural extracts are favored for their minimal side effects. AgNPs have gained prominence for their antibacterial potential, disrupting bacterial cell walls regardless of drug resistance [10]. Their tissue permeability, biocompatibility, and effectiveness make them potential solutions for root canal disinfection [11]. This research highlights *A. indica* AgNPs' and TSC AgNPs' efficacy against *E. faecalis*, notorious for resisting standard disinfectants in root canal infections. While biosynthesized AgNPs from *A. indica* in root canal disinfection are limitedly studied, this underscores their antibacterial potency against *E. faecalis*, offering promising alternatives for root canal therapy [12]. *A. indica *AgNPs showed the highest inhibitory impact on *E. faecalis*, followed by chemically synthesized AgNPs [13]. MBC testing revealed *A. indica* AgNPs' superior efficacy, especially at 100 μL concentration, with lower CFU counts, emphasizing their effectiveness in inhibiting bacterial growth [14,15].

Kim et al. synthesized AgNPs from silver nitrate and sodium borohydride, demonstrating substantial growth inhibition of *Escherichia coli* and *Staphylococcus aureus* from 0.2 nM to 33 nM. At 33 nM, their efficacy matched the positive control, surpassing gold nanoparticles against *E. coli *[16]. Owaid et al. used AgNPs derived from *Pleurotus cornucopiae var. citrinopileatus*, exhibiting enhanced suppression of pathogenic *Candida* species at 60 µg/well. Pure extract at 20-40 µg/well showed limited efficacy, with the antibacterial effect attributed to electrostatic interactions between silver ions and microbial cell membranes, facilitating cell penetration and death [17].

Allahverdiyevet al. highlighted the potential of AgNPs in reducing antibiotic toxicity to human cells while restoring their effectiveness against resistant germs [18]. Vijay Kumar et al. showcased diverse AgNPs' efficacy; *Boerhavia diffusa* extract-derived AgNPs effectively targeted *Flavobacterium branchiophilum* in fish, while *Piper longum* fruit extract-derived AgNPs exhibited cytotoxic effects on MCF-7 breast cancer cells, indicating potential in cancer therapy with added antibacterial and antioxidant properties [19]. Latha et al. utilized *Adathoda vasica* leaf extract for AgNPs, demonstrating antibacterial efficacy against *Vibrio parahaemolyticus* in agar media, suggesting promise in seafood-related disease treatment without toxicity to *Artemia nauplii* [20].

In a study by Vestby et al.,* *significant biofilm-related human infections, attributed to pathogens like *S. aureus, E. faecalis*, and *Pseudomonas aeruginosa*, show promising susceptibility to *A. indica* [21]. Notably, *A. indica* extracts, particularly an *A. indica* leaf ethanolic extract, exhibited inhibitory effects on *S. aureus* and MRSA biofilm adherence according to Quelemes et al. [22]. Guchhait et al. concluded that ripe *A. indica* seed extracts also exhibited antibiofilm activity against *S. aureus *and *Vibrio cholerae *[23]. *A. indica*-derived phytochemicals, like catechin, present potential for biofilm eradication and quorum-sensing prevention by Lahiri et al.* *[24]. According to Wylie et al., methanolic *A. indica *leaf extract and nimbolide exhibited efficacy against *Helicobacter*​​​​​​​* pylori* biofilms in vitro [25]. These studies highlight AgNPs' diverse applications across antibacterial, anticancer, and disease-specific treatments.

*A. indica* AgNPs show promise as root canal irrigants, offering potent antibacterial action with low cytotoxicity. Yet, concerns linger about potential periapical tissue effects and dentin discoloration. More in vivo and in vitro studies are crucial for clinical application validation. This study highlights AgNPs' efficacy against *E. faecalis* but focuses solely on in vitro experiments, necessitating clinical validation. Limited bacterial strain exploration and cytotoxicity assessment pose challenges. Exploring diverse plant extracts for AgNPs' synthesis and addressing delivery, stability, and long-term effects are vital for practical endodontic use. Overcoming these limitations will unlock AgNPs' clinical potential in endodontic therapy.

The present study presented herein contributes valuable insights into the synthesis and characterization of AgNPs using *A. Indica* extract and TSC, along with an assessment of their cytotoxicity and antimicrobial efficacy against *E. faecalis*. However, certain limitations warrant consideration for a comprehensive understanding and interpretation of the findings. First, while the study diligently outlines the experimental procedures for AgNPs' synthesis and characterization using various analytical techniques such as UV-vis spectroscopy, SEM, and XRD, the absence of comparative analyses with standard methods or commercially available AgNPs limits the assessment of the novelty and efficacy of the synthesized nanoparticles. Moreover, the study primarily focuses on in vitro assays to evaluate cytotoxicity and antimicrobial activity, thereby overlooking potential complexities associated with in vivo environments, such as tissue interactions and systemic effects, which are crucial for translational research in medical applications.

To enhance the robustness and translational relevance of the study, several improvements could be implemented. First, incorporating comparative analyses with commercially available AgNPs or standard antimicrobial agents would provide a benchmark for assessing the efficacy of the synthesized nanoparticles. Additionally, conducting in vivo studies, such as animal models or ex vivo tissue cultures, would offer insights into the nanoparticles' behavior in more physiologically relevant settings, enabling a more comprehensive evaluation of their safety and efficacy. Furthermore, expanding the scope of antimicrobial testing to include a broader range of bacterial strains or biofilm models would better reflect the clinical diversity of microbial infections encountered in endodontic practice. Finally, incorporating cytotoxicity assessments on human cell lines or primary cultures would provide valuable insights into the nanoparticles' biocompatibility and potential for clinical applications.

## Conclusions

This study highlights the promising antibacterial potential of *A. indica *AgNPs and TSC AgNPs against *E. faecalis* for endodontic use. *A. indica* AgNPs demonstrated superior activity over TSC AgNPs, showing lower cytotoxicity initially. Yet, comprehensive toxicity studies and in vivo models are needed for safety confirmation. Broader microbial exploration and clinical applicability assessment are crucial. Caution is warranted concerning tissue impact and long-term effects before clinical implementation. While *A. indica* AgNPs hold promise as an endodontic disinfectant, further research is essential to ensure their safety and effectiveness in clinical practice.
